# Effect of preoperative pulmonary artery pressure on the prognosis of end-stage heart failure patients after heart transplantation

**DOI:** 10.1186/s13019-023-02253-x

**Published:** 2023-04-17

**Authors:** Wei Zhou, Zhen Du, Yun Tao, Guan-xin Zhang, Zhi-yun Xu, Lin Han, Shao-lin Ma, Dao-xi Hu, Xing-li Fan, Bai-ling Li

**Affiliations:** 1 Department of Critical Care Medicine, Shanghai East Hospital, Shanghai, 200120 China; 2 Department of Cardiovascular Surgery, The First Affiliated Hospital of Naval Medical University, Shanghai, 200433 China; 3 Department of Medical Imaging, Army 75 Group Military Hospital, Dali, 671000 China

**Keywords:** End-stage heart failure, Pulmonary artery pressure, Heart transplantation

## Abstract

**Objective:**

To evaluate the effect of preoperative pulmonary artery pressure on perioperative outcome of end-stage heart failure patients undergoing heart transplantation.

**Methods:**

Retrospective analysis was undertaken on the clinical data of patients receiving heart transplantation in the Department of Cardiovascular Surgery of our hospital from March 2017 to March 2022. A ROC curve analysis was developed between mean pulmonary artery pressure (mPAP) and postoperative mortality using mPAP as diagnostic criteria. Patients were divided into groups based on this threshold to determine the best mPAP threshold value for predicting postoperative nosocomial mortality, and the differences in preoperative and intraoperative data, postoperative complications, and clinical prognosis of patients in the two groups were compared. Patients were followed up to draw the survival curve of patients in the two groups.

**Results:**

The study enlisted the participation of 105 patients. ROC curve research revealed that preoperative pulmonary artery pressure was substantially linked with death following heart transplantation, with mPAP = 30.5mmHg being the best threshold. The group with mPAP ≥ 30.5mmHg had a greater incidence of postoperative ECMO support (28.2% vs. 10.6%, P = 0.021) and a higher incidence of in-hospital mortality (15.4% vs. 1.5%, P = 0.019) than the group with mPAP < 30.5mmHg. The postoperative survival rates of 105 patients were 91.3%, 88.7%, 81.6%, and 77.5% at 1, 2, 3, and 4 years, respectively, however, there was no significant difference between the two groups of patients in the postoperative intermediate-far survival rate (P = 0.431).

**Conclusions:**

Preoperative pulmonary artery pressure in patients with end-stage heart failure is intimately correlated with perioperative prognosis of heart transplant recipients. The optimal cut-off mPAP value in predicting perioperative prognosis of heart transplant recipients is 30.5mmHg. In the high mPAP group, perioperative ECMO support rate and perioperative mortality rate are high, which do not affect the medium and long-term prognosis of the recipients undergoing heart transplantation.

## Introduction

The most successful treatment for end-stage heart failure is heart transplantation [1]. More and more Cardiovascular Surgery institutes in China are performing heart transplantation procedures with a better postoperative prognosis as perioperative care concepts in cardiac surgery evolve, surgical techniques mature, and donor heart protection techniques improve [2]. Related studies have shown that preoperative pulmonary hypertension is a contraindication for orthotopic heart transplantation and an important risk factor for postoperative right ventricular failure [3]. Most individuals with end-stage heart failure develop pulmonary hypertension of varied degrees due to the disease’s relatively protracted course. As a result, preoperative pulmonary arterial pressure should be reversibly evaluated in order to optimize patient outcomes and maintain right heart function after surgery [4]. This study focused on retrospectively analyzing the clinical data of patients undergoing heart transplantation in our hospital, analyzing the relationship between preoperative pulmonary artery pressure and postoperative complications and in-hospital mortality, and summarizing clinical experience in order to reduce the incidence of postoperative complications and improve the survival rate and postoperative quality of life of patients after heart transplantation.

## Methods

### Patients

A retrospective analysis was performed on the clinical data of 115 patients undergoing heart transplantation in the Department of Cardiovascular Surgery of our hospital from March 2017 to March 2022. 2 patients under 18 years of age, 3 patients with preoperative placement of IABP and 5 patients assisted by ECMO were excluded, a total of 105 patients were enrolled in the study, including 79 patients with dilated heart disease, 9 patients with ischemic cardiomyopathy, 12 patients with valvular heart disease, and 5 patients with other diseases. All recipients complete the preoperative examination of heart transplantation, register on the national organ transplantation platform, wait for a suitable donor, and undergo surgery with the approval of the ethics committee; During the operation, anesthesia combined with inhalation and intravenous injection was used, and the operation was performed under CPB. After the operation, cyclosporine or tacrolimus + metecophenol + methylprednisolone was given to prevent rejection, and routine treatment was received in ICU.

### Definition and grouping

According to the diagnosis and treatment guidelines for pulmonary hypertension, mean pulmonary artery pressure (mPAP) > 20mmHg was taken as the diagnostic standard [5]. mPAP was mainly used as the research index in this study. Right heart catheterization (RHC) was performed for patients who need to receive transplantation. During the waiting period, patients will be re-evaluated every 3–6 months to check cardiac function and pulmonary artery. mPAP used in the study was the last measured value before surgery. ROC curve analysis of mPAP and postoperative mortality was developed to determine the optimum critical mPAP value to predict postoperative nosocomial mortality. Patients were divided into groups based on the best threshold, and the differences in preoperative and intraoperative data, postoperative complications and clinical prognosis were compared between the two groups. The postoperative survival rate and quality of life of the patients were continuously followed up to draw the survival curve of the two groups of patients.

### Statistical analysis

Continuous variables were expressed by mean ± standard deviation, and classified variables were expressed by percentage. T-test or Wilcoxon rank-sum test was used to analyze the difference between the two groups of continuous variables, and the Chi-square test was used to analyze the difference between the two groups of classification variables. ROC curve analysis was used to evaluate the mPAP threshold for predicting postoperative nosocomial death, and the value of mPAP in predicting postoperative nosocomial death was evaluated by calculating the area under ROC curve. The Kaplan-Meier method was used to calculate the overall survival rate between the two groups, and the log-rank test was used to compare the differences between groups.

## Results

**Fig. 1:**
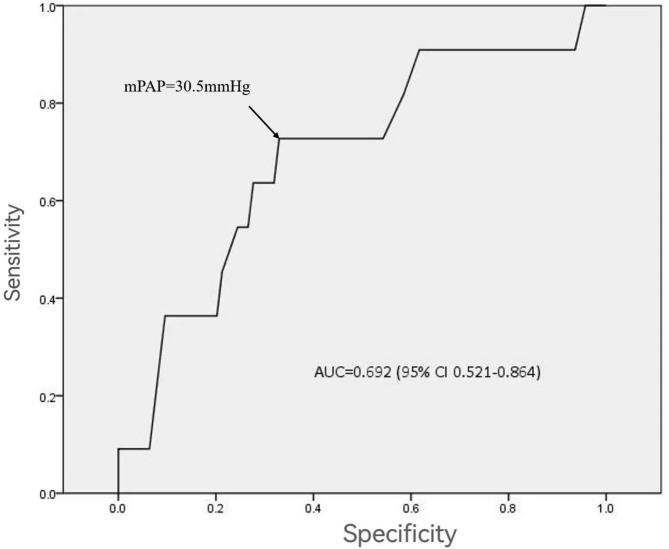
ROC curve: The ROC curve showed that mPAP = 30.5mmHg was the best cut-off value for predicting postoperative nosocomial death

ROC curve analysis showed that preoperative pulmonary artery pressure was substantially linked with death following heart transplantation (AUC = 0.692(95%CI 0.521–0.864)), with mPAP = 30.5mmHg being the best threshold. *(*Fig. 1*)*. Therefore, according to this critical value, patients were divided into mPAP < 30.5mmHg group and mPAP ≥ 30.5mmHg group.

### Baseline and preoperative characteristics

A total of 105 patients were included in the study, including 66 patients in mPAP < 30.5mmHg group and 39 patients in mPAP ≥ 30.5mmHg group. Patients in mPAP ≥ 30.5mmHg group had higher recipient body weight (72 ± 13.4 vs. 64 ± 11.8, P = 0.015) and lower donor/recipient body weight ratio (1.0 ± 0.16 vs. 1.1 ± 0.26, P = 0.011). A higher proportion of patients had a history of coronary intervention (17.9% vs. 0%, P = 0.001) and a longer preoperative transplant wait time (81 ± 98.9 vs. 67 ± 124.4, P = 0.011). There were no significant differences in other indicators such as male proportion, diabetes history and etiological classification. (Table 1).

**Table 1:** Baseline and preoperative characteristics

Variable	mPAP < 30.5mmHg (n = 66)	mPAP ≥ 30.5mmHg (n = 39)	P value
Men(%)	50(75.8)	35(89.7)	0.815
Age(years)	48 ± 14.6	50 ± 12.3	0.550
Recipient weight(Kg)	64 ± 11.8	72 ± 13.4	0.015
Weight of donor(Kg)	70 ± 10.4	71 ± 10.8	0.340
Body weight ratio (donor/recipient)	1.1 ± 0.26	1.0 ± 0.16	0.011
Diabetes(%)	4(6.1)	3(7.7)	1.000
Hypertension(%)	4(6.1)	6(15.4)	0.219
Stroke(%)	0	1(2.6)	0.371
Previous surgical history			
Valve surgery(%)	4(6.1)	2(5.1)	1.000
Coronary intervention(%)	0	7(17.9)	0.001
ICD(%)	3(4.5)	4(10.3)	0.466
Congenital heart disease surgery(%)	2(3.0)	0	0.529
Coronary artery bypass grafting(%)	1(1.5)	0	1.000
Left ventricular assist device(%)	1(1.5)	0	1.000
Transcatheter radiofrequency ablation(%)	1(1.5)	0	1.000
Cause			
Dilated cardiomyopathy(%)	51(77.3)	28(71.8)	0.530
Ischemic cardiomyopathy(%)	4(6.1)	5(12.8)	0.404
Valvular heart disease(%)	7(10.6)	5(12.8)	0.730
Congenital heart disease(%)	1(1.5)	0	1.000
Restrictive cardiomyopathy(%)	1(1.5)	1(2.6)	1.000
Heart tumor(%)	1(1.5)	0	1.000
Myocardial amyloidosis(%)	1(1.5)	0	1.000
Waiting Time(day)	67 ± 124.4	81 ± 98.9	0.011
Mean pulmonary artery pressure(mmHg)	21 ± 5.4	39 ± 6.5	0.000
Laboratory examination			
Hemoglobin(g/L)	124 ± 10.4	126 ± 9.6	0.230
Creatinine(µmoI/L)	88 ± 15.4	92 ± 13.8	0.451
pro-NBP(ng/L)	286 ± 138.2	298 ± 134.6	0.466
Leukocyte(10^9^/L)	8.9 ± 2.4	8.6 ± 3.4	0.364

ICD: Implantable cardioverter defibrillator

### Surgery and postoperative characteristics

Compared with mPAP < 30.5mmHg group, mPAP ≥ 30.5mmHg group had a greater probability require ECMO support after surgery (28.2% vs. 10.6%, P = 0.021) and a higher in-hospital mortality (15.4% vs. 1.5%, P = 0.019). There were no abnormalities in the intraoperative extracorporeal circulation time, aortic occlusion events, and circulation assistance time, and no abnormalities in the postoperative IABP support ratio, mechanical ventilation time, and ICU retention time. (Table 2).

**Table 2:** Intraoperative and postoperative characteristics

Variable	mPAP < 30.5mmHg (n = 66)		mPAP ≥ 30.5mmHg (n = 39)		P value
Intraoperative data					
Combined heart and kidney transplantation(%)	2(3.0)	0		0.529	
CPB time(min)	139 ± 41.8	150 ± 38.2		0.127	
ACC time(min)	43 ± 10.6	45 ± 10.4		0.193	
Circulation assist time (min)	89 ± 43.1	96 ± 41.1		0.321	
Donor cold ischemia time (min)	181 ± 130.9	189 ± 121.7		0.486	
Postoperative data					
IABP(%)	10(15.2)	12(30.8)		0.057	
IABP assist time(h)	172 ± 83.3	171 ± 68.8		0.772	
ECMO(%)	7(10.6)	11(28.2)		0.021	
ECMO assist time(h)	167 ± 80.9	157 ± 57.3		0.500	
Mechanical ventilation time(h)	41 ± 87.5	78 ± 139.0		0.080	
ICU stay(day)	13 ± 6.2	13 ± 6.3		0.981	
Moderate and severe tricuspid regurgitation(%)	15(22.7)	10(25.6)		0.735	
Death(%)	1(1.5)	6(15.4)		0.019	

CPB: cardiopulmonary bypass; ACC: aortic cross-clamp; IABP: Intra-aortic balloon pump; ECMO: Extra-Corporeal Membrane Oxygenation; ICU: Intensive care unit

### Follow-up

**Fig. 2:**
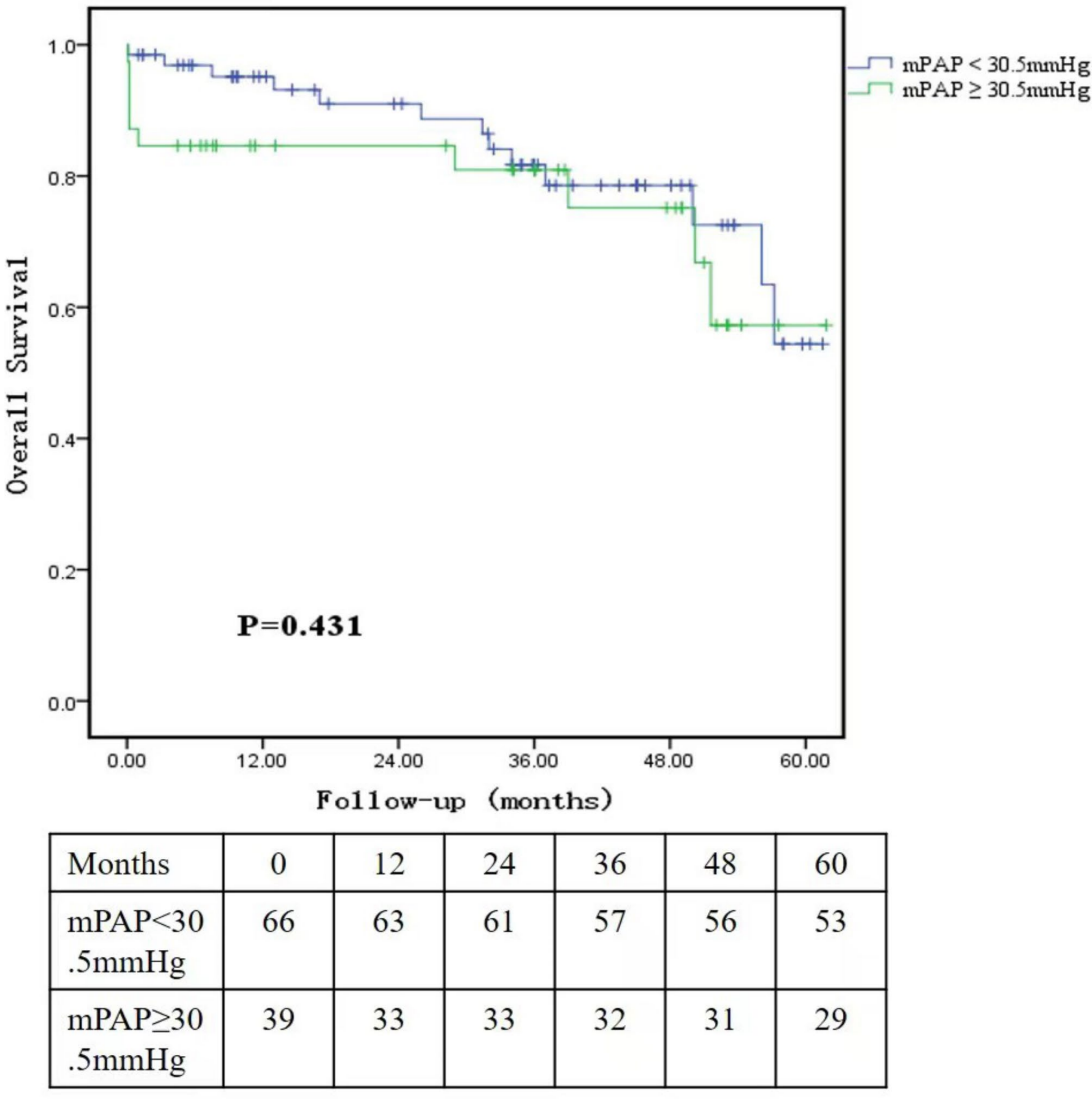
Survival curve: Kaplan-Meier survival curve analysis of patients in the two groups during follow-up period

The follow-up period was 26 ± 20.5 months with100% rate. The survival rates of the 105 patients at 1, 2, 3, and 4 years after surgery were 91.3%, 88.7%, 81.6%, and 77.5%, the survival rates of low mPAP group at 1, 2, 3, 4 years after surgery were 98.5%, 91.0%, 81.7%, 78.6%, and high mPAP group were 84.6%, 80.9%, 80.9%, 75.2%. However, Kaplan-Meier survival curve analysis of the follow-up results of the two groups showed that there was no significant difference between the two groups in the postoperative intermediate-far survival rate (P = 0.431). (Fig. 2).

## Discussion

End-stage heart failure is the cause of death or need for heart transplantation [6]. However, such patients tend to have a particularly long course of disease with pulmonary hypertension in the later stages [7, 8]. While preoperative pulmonary hypertension is one of the risk factors for early right ventricular dysfunction and perioperative death after heart transplantation, so preoperative assessment of pulmonary artery pressure is crucial [9]. The criteria of threshold guidelines for pulmonary hypertension used to define convoys for heart transplantation are also inconsistent, and pulmonary artery systolic blood pressure is mainly used, Moreover, it needs to be combined with pulmonary vascular resistance (PVR) to evaluate outcomes in heart transplant patients [10]. According to 2015 ESC/ERS guidelines [11], the main evaluation index for the definition of pulmonary artery hypertension is pulmonary artery mean blood pressure, so we used mPAP as the main object of this study. To study its influence on perioperative prognosis after transplantation. ROC curve analysis showed that preoperative pulmonary artery pressure was significantly correlated with death after heart transplantation. Preoperative mPAP = 30.5mmHg was the best cut-off value for predicting postoperative nosocomial death. The group with mPAP ≥ 30.5mmHg had a higher proportion of postoperative ECMO assistance and higher nosocomial death.

End-stage heart failure patients mainly with left heart systole and diastole dysfunction, which is one of the most common cause of pulmonary artery pressure in left heart disease, its pathological physiological characteristics of left ventricular filling pressure, secondary left atrial remodeling and pulmonary vein circumfluence suffocate, further lead to pulmonary vein pressure, with the progression and pressure conduction, Pulmonary artery endothelial dysfunction and reactive vasoconstriction, neuroendocrine cell activation and inflammatory changes, pulmonary vascular remodeling, cause the pulmonary arterial hypertension (PAH) [12], When blood from the transplanted donor heart is injected into the pulmonary circulation with high pressure and resistance, cardiac output is reduced and higher resistance needs to be overcome [13], which may easily lead to acute right ventricular failure. Relevant literature reports that the donor heart is usually unable to withstand the right cardiac afterload of 50mmHg, and when the pulmonary artery systolic pressure exceeds 55-60mmHg, the probability of right heart failure increased significantly [14]. For patients with preoperatively existing pulmonary hypertension and mPAP ≥ 30.5mmHg, close monitoring should be conducted in the early postoperative period, We routinely use diuresis to reduce cardiac preload, while levosimendan or sildenafil were used to reduce the pulmonary pressure of patients. In some patients, pulmonary artery pressure can not be effectively controlled and gradually increases, leading to acute right heart failure. When pulmonary hypertension is difficult to control and accompanied by right cardiac insufficiency, mechanical assistance devices such as IAPB and EMCO should be used as soon as possible.

According to the follow-up data, patients with mPAP ≥ 30.5mmHg had a high postoperative in-hospital mortality, but there was no significant difference in the long-term mortality, which was also consistent with the United States UNOS database research report [15]. Nosocomial death after heart transplantation is mainly caused by early postoperative complications, Mainly including graft dysfunction, rejection, infection, et al., while Late complications mainly include graft vasculopathy, malignant tumor and kidney function dysfunction [16]. Preoperative pulmonary hypertension is more likely to influence the early prognosis, but the long-term death after heart transplantation is related to multiple factors such as rejection monitoring, immunosuppression program and community infection. Therefore, it is necessary to fully follow up each patient regularly and evaluate the relevant risk indicators, so as to customize the treatment regimen beneficial to the patient, such as immunosuppression, so as to improve the medium-long-term survival rate.

This study has some limitations related to its retrospective design and the fact that all data was generated by a single center. Due to its inherent limitations, it is impossible to exclude all confounding factors, especially those factors that have a significant impact on prognosis such as anti-rejection therapy after heart transplantation; Moreover, the conclusions may be influenced by this center’s practice standards, thus, multi-center studies should be carried out to obtain further insights.

## Conclusions

Preoperative pulmonary artery pressure in patients with end-stage heart failure is intimately correlated with perioperative prognosis of heart transplant recipients. The optimum cut-off mPAP value in predicting perioperative prognosis of heart transplant recipients is 30.5mmHg. In the high mPAP group, perioperative ECMO support rate and perioperative mortality rate are high, which do not affect the medium and long-term prognosis of the recipients undergoing heart transplantation.

## Data Availability

The datasets used during the current study are available from the corresponding author on reasonable request.
